# Measurement of perceived needs in humanitarian contexts using the HESPER scale: a scoping study with reflections on the collaboration between researchers and humanitarian actors

**DOI:** 10.1186/s13031-022-00478-6

**Published:** 2022-08-26

**Authors:** Karin Hugelius

**Affiliations:** Faculty of Medicine and Health , Örebro University, Örebro, Sweden

**Keywords:** Needs assessment, Humanitarian emergencies, Mental health, Disaster, Humanitarian aid

## Abstract

**Background:**

Needs assessment is one of the fundamental humanitarian responses to sudden-onset or long-lasting emergencies. The Humanitarian Emergency Settings Perceived Needs Scale (HESPER)/ HESPER Web are valid scales for identifying perceived needs among humanitarian or disaster-affected populations, both in humanitarian practice and in science. This scoping review aimed to determine the scientific use of HESPER or HESPER Web, report on previously published perceived needs in humanitarian emergencies, and discuss how scientific and humanitarian actors can work together in a partnership in needs assessment in humanitarian settings.

**Results:**

In all, eight papers were found in which the HESPER or HESPER Web had been used in conflict- or post-conflict settings or natural disasters. The study samples varied from 85 to 1000 participants (mean 440). The mean number of perceived needs in all studies was 8, varying from 4.25 to 12.18. The top three needs varied in all the studies. A high number of perceived needs was associated with mental health problems. No paper has reported on how the assessment outcomes were shared between the researchers and humanitarian actors.

**Conclusion:**

Inventorying the needs from the perspective of the affected population is important to tailor the response to each humanitarian emergency. The HESPER scale and the HESPER Web are multisectoral tools that can be used to take inventory of the perceived needs and indicate the mental health problems that arise in conflict-ridden and natural disaster contexts. It is essential that results from a scientific needs inventory are shared with adequate humanitarian stakeholders to not only facilitate a proper response, but also to foster a closer collaboration between scientists, humanitarians, and the affected population. Doing so would increase the development and use of evidence in practice when providing humanitarian aid.

## Introduction

Needs assessment is one of the fundamental responses to addressing humanitarian crises [1]. Reliable and relevant information on needs is crucial to make sound decisions on what kind of relief is needed and to whom, where, and when it should be delivered. However, such information is rarely available to decision makers and the formal needs assessments required to generate such reliable data are sometimes still lacking [2]. Previously it has been common that humanitarian agencies tended to focus on needs assessment within a specific field, often related to their own programmes, such as water and sanitation or mental health, rather than getting a comprehensive picture of the needs in a broader population [2]. The same challenge is evident in research studies conducted in humanitarian settings. However, methods for multi sectorial needs assessments are available from example from the public health information systems toolkit provided by the Global Health Cluster [3]. The Humanitarian Programme Cycle (HPC) is another tool to prepare for, manage, and deliver a humanitarian response, which has been provided by the United Nations Office for the Coordination of Humanitarian Affairs [4]. It consists of tools and recommendations to coordinate the five phases in a humanitarian response, with needs assessment and analysis occupying the first phase and eventually leading to the formulation of a humanitarian needs overview that lay the basis for a humanitarian response plan [4]. The programme offers several tools and templates for conducting needs assessments and reporting the findings. One of the suggested tools is the Humanitarian Emergency Settings Perceived Needs Scale (HESPER) [5, 6]. The original HESPER collected data through face-to-face interviews, asking the respondents to state whether a certain need was perceived as serious and offering the respondent to prioritise their most significant perceived needs. In addition to the original scale, a web-based version (HESPER Web) has been developed [7]. The HESPER/ HESPER Web scale consists of 26 items covering physical, psychological, and social determinants of health and well-being and provides a picture of the perceived needs from the perspective of the affected population. The psychometrics and alternate forms reliability between the two versions have been reported elsewhere [8].

Another challenge is related to the inclusion of affected populations in humanitarian relief, as well as in science [9]. Such perspectives still seem rare in both humanitarian practice and research [10], as predicting needs in humanitarian contexts is difficult [11]. The HESPER scale/ HESPER Web was developed and evaluated specifically for use both by humanitarian actors and in research. However, little is known about the use of the scale, or how information on the use has been shared with humanitarian actors in the field. Therefore, the aim of this paper was to determine the scientific use of the HESPER or HESPER Web, report on the perceived needs in humanitarian emergencies, and discuss how scientists and humanitarian actors can work together in a partnership in needs assessment activities in humanitarian settings.

## Methods

A scooping study in accordance with the methodology suggested by Arksey and O’Malley was conducted with the purpose of summarising and disseminating the research findings [12]. The five suggested stages were followed.

### Stage 1. Identifying the research question

The research questions for this study were the following:

1. In what contexts and study populations, and with what study designs has the HESPER scale been used in scientific studies?
2. What needs have been reported when studying the perceived needs in populations affected by humanitarian emergencies?
3. How were potential collaborations or information on the needs assessment results shared between the researchers and humanitarian stakeholders involved in the study context described in the paper?

### Stage 2. Identifying relevant studies

A structural search of the PuBMed and Web of Science databases was conducted on January 17, 2022. The search terms used and the matches found are presented in Table 1. To be included, a paper had to be published in English during the last 20 years and have used the HESPER scale in any version or language to identify perceived needs. All kinds of scientific publications, such as original studies, case reports, and conference papers, were included. Exclusion criteria were papers reporting strictly psychometric results or data on the HESPER scale itself.

**Table 1:** Overview of database searches, search terms, and matches

	Search terms	Number of records
PubMed	S1: [The Humanitarian Emergency Settings Perceived Needs Scale]	9
	S2: [“HESPER” AND humanitarian]	9
	S3: [(humanitarian) AND (needs assessment)]	299
	Total	81
Web of Science	S1: [The Humanitarian Emergency Settings Perceived Needs Scale]	9
	S2: [“HESPER” scale]	1
	Total	10
Total for all searches	N	32

### Stage 3. Study selection

**Fig. 1:**
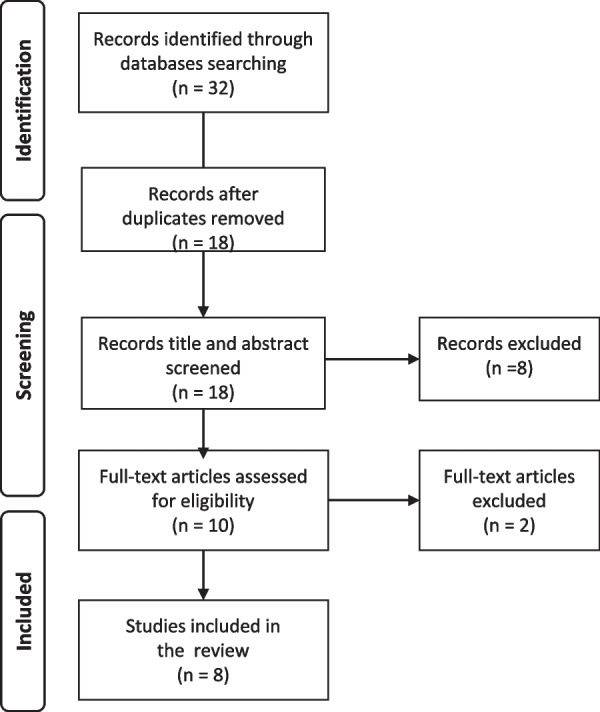
Flowchart of the selection process

All studies identified in the database search were assessed for the inclusion criteria, starting with the title and abstract. A full-text reading was then performed for all papers not yet being excluded (see Fig. 1). After the relevant papers were identified from the database search, a manual search of the reference lists was conducted. No further study in need of inclusion was identified.

### Stage 4. Charting the data

Information on authors, year of publication, study design, setting, country of data collection, sampling method, study sample, top three needs reported in the study, and additional results with relevance for either the research question on the use of HESPER or the needs reported were charted manually (see Table 2). These data were subject to the core analysis of the study [12].

**Table 2:** Overview of included studies, reported needs and findings

References	Study design	Setting	Country	Study sample*	Sampling method	Top three needs reported	Mean of needs	Other findings	Other instruments used
Ayazi et al. [14]	Cross-sectional	Conflict or post conflict	South Sudan	N = 464	multistage random cluster sampling	drinking water, alcohol, and drug use in the community and access to sanitation facilities	12.18, CI (11.57 to 12.80)	Higher level of perceived needs significantly predicted psychological distress	General Health Questionnaire (GHQ 12) and Short Form Health Survey (SF-12)
Bapolisi et al. [16]	cross-sectional	Conflict or post conflict	Uganda	N = 387	Quota and stratified sampling	Distress Care for family member Healthcare	N/A	PTSD was positively associated with stress	International Neuropsychiatric Interview
Da Silva et al. [18]	Cross sectional	Hurricane	Costa Rica	N = 1000	N/A	Health problem Distress Lack of adequate help	N/A	Needs assessment is fundamental for a proper response after a hurricane	None
Falb et al. [15]	Cross sectional	Conflict or post conflict	Syria	N = 214	N/A	N/A	12.2 (S.D.: 3.4; range 2–20)	Mean of perceived needs was associated with currently being displaced and reporting some form of disability. Mental health needs of women in conflict areas needs to be further addressed	Nine-item Patient Health Questionnaire (PHQ-9), Household Food Insecurity Access Scale (HFIAS), Washington Group on Disability Statistics Short Set Questions, intimate partner violence
Jordans et al. [13]	Cross sectional	Conflict or post conflict	Jordan, Nepal	Jordan; n = 269, Nepal, n = 269), total N = 538	Multistage cluster sampling	Not stated	10.61 SD (5.88) and 8.10 (4.64)	Indirect effects of trauma exposure on distress via current perceived unmet needs were found	GHQ-12, Composite International Diagnostic Interview (CIDI)
Kane et al. [17]	Cross sectional	Earthquake	Nepal	N = 513	stratified multi-stage cluster sampling	Shelter, Distress, Income/ livelihood	9.73 to 3.27	A greater number of perceived needs was associated with higher odds of depression, anxiety and PTSD	Hopkins Symptom Checklist-25, PTSD Checklist-Civilian, hazardous alcohol use (AUDIT-C), Assessment Schedule of Serious Symptoms in Humanitarian Settings (WASSS) Composite International Diagnostic Interview
Hugelius et al. [7, 19]	Cross sectional	Conflict or post conflict	Sweden	N = 85	Purposive sampling	income or livelihood, separation from loved ones, being displaced from home	4, (SD 2.71)	Addressing people’s current perceived needs should be considered in health care systems that cater to immigrants	Qualitative interviews
Hugelius et al. [8]	Cross sectional	Conflict or post conflict	Kenya	N = 320	Purposive sample	Income or livelihood, Too much free time, Law and justice in your community	4.52 (SD 3.2, range 1–15)	HESPER Web was found to be reliable and usable for assessing perceived needs in humanitarian emergencies	None

^*^If the study had several aims or parts, the study sample used to assess perceived needs was reported in this table

### Stage 5. Collating, summarising, and reporting the results

An overview of the papers included is presented in table form (Table 2).

## Results

In all, eight scientific papers using the original HESPER scale (n = 6) or the HESPER Web scale (n = 2) were identified.

### Study contexts

All the papers had a cross-sectional design. Most of the studies (n = 7) had been carried out in conflict or post-conflict contexts [7, 8, 13–16]. Other studies were conducted four months after an earthquake [17] and six to 12 months after a hurricane [18]. Three studies were conducted in African countries (Uganda, [16], South Sudan [14], and Kenya [8]), two in Asia (Nepal [13, 17]), two in the Middle East (Jordan [17] and Syria [15]), and one in Europe (Sweden [7]).

### Study samples

Half of the studies had used randomised or quota study sampling based on lists [13, 16], the random-walk method [13, 15, 16], or another method [14, 17] (see Table 2). The remaining four studies used a non-randomised convenience sample [8, 19] or did not state whether any randomisation had been used [15, 18]. The study sample size varied from 85 to 1000 study participants (mean 440, median 425), leading to a total of 3521 study participants being covered in this review. In two studies, the study sample was a part of a specific population (e.g., females participating in a cash transfer programme [15] or people 65 or older [18]).

### Reported needs

The mean of the total reported needs was eight (mean 8.0, median 8.8, varying from a mean of 4.25 to 12.18). The top three needs varied in all studies reporting on perceived needs (see Table 2). Psychosocial needs, such as distress, separation from loved ones, or care for family members, were more frequently reported as the top three needs than physical needs, such as clean water or shelter.

### Relationship between perceived needs and mental health

Several studies [13–17] used additional scales or instruments. Most commonly, the HESPER scale was combined with the General Health Questionnaire (GHQ) [13, 14]. Also, several other instruments covering mental or psychosocial health were often combined with the HESPER scale [13–17] to determine significant relationships between well-being or mental health conditions and perceived needs. Several studies presented evidence on the relationships between (1) perceived needs and psychological distress [14], (2) posttraumatic stress disorder (PTSD) and the reporting of distress [16], and (3) perceived unmet needs and distress [13]; another study reported higher odds of depression, anxiety, and PTSD among people reporting a high rate of needs [17].

### Collaboration between researchers and humanitarian actors

None of the included papers directly reported whether the perceived needs in the study population had been shared with any humanitarian actor operative in the specific emergency covered by the study. However, some of the papers indicated collaborations of some kind between scientists and humanitarian actors in the affiliations or acknowledgements of the paper [6, 13, 15].

## Discussion

This scoping review has shown that the HESPER scale has been used in scientific studies covering different humanitarian contexts to report on perceived needs, as well as to demonstrate a significant relationship between perceived needs and mental health in humanitarian populations.

Given the disparity of the needs considered to be a top priority in the different populations and humanitarian emergencies and the variation in the total number of perceived needs, this study emphasises the importance of involving the affected population and relying on primary data when estimating needs. Analysis of secondary data should rely on valid primary sources where data is collected using valid tools and directly from the affected population. This is an important finding, especially in humanitarian aid responses, when needs assessments relying on secondary data are not uncommon [1]. The findings also indicated that psychological needs were reported more frequently than physical needs. Possible explanations for this could be that psychosocial needs were actually either more frequent and emphasised among the study participants compared to physical needs, that psychosocial needs were underestimated by the humanitarian actors and therefore not met, or that physical needs had already been satisfied when conducting the inventory. The disparity between reported top priority needs also emphasises the importance of using a multisectoral tool to take inventory of needs, especially when conducting research in the early phase of a humanitarian emergency. One perspective that cannot be answered by the studies included in this review is the reasons why the perceived needs could not be met. This is a question that deserved further attention.

Conducting research in humanitarian contexts entails several methodological challenges. Given certain practical realities, such as a lack of baseline and personal data, organised registers, infrastructure, population movements, security threats, and dynamic environments, innovative initiatives might be necessary to conduct research in humanitarian emergencies [20, 21]. One challenge pertains to the difficulty of recruiting a representative study sample [21, 22]. Within this review, strategies to select representative study samples included different quotas or two-stage cluster sampling strategies based on making lists or walking from household to household. Such strategies have been criticised, however, since they do not take dynamic movements or changes in the population, which are common in humanitarian contexts, into consideration [23]. The use of satellite pictures has been suggested to ease two-stage clustering [24], but such information is not available in all situations. The use of the HESPER or similar tools does not solve the problem of sampling strategies. However, a valid sampling strategy might not be the same as a practically valid sample. A critical discussion on what is an acceptable sample and sampling strategy, given the practical circumstances a humanitarian context implies, is a sound basis for making operational decisions and priorities and can also be considered necessary for compliance with ethical and safety practices [25]. This review included both the original HESPER and HESPER Web. When choosing which tool to use, the context, availability of internet, population, security, and possibilities for physical access must be taken into consideration. Ensuring confidentiality and a safe storage of data is essential, both for face to face interviews and digital data collections.

Asking a person affected by a humanitarian crisis about their current need may raise expectations that the needs will be met in the near future. None of the included papers specifically reported that they had shared their results with any humanitarian actor or other stakeholder on site. If this is the actual circumstances, this is problematic since a lack of response to the needs reported may lead to distrust between the affected population, researchers, and humanitarian actors at the site [22, 26]. However, it is possible that the collaboration mentioned in the studies in data collection also included data sharing and that the results were shared to influence the immediate response, even if this is not clearly stated. If not, this is an issue that needs further attention. Early information sharing of gathered data has been found essential to promoting an interest in scientific knowledge in humanitarian fieldwork [22]. Also, scientists need the perspectives of humanitarian field workers to validate and interpret their results [27]. Therefore, it must be recommended that the results from needs assessment be shared between scientists and humanitarians and used to influence emergency response.

Given the limited level of scientific evidence in humanitarian practice [28, 29], the use of validated instruments offers some possibilities to compare and discuss trends and, in the future, maybe even to generalise need estimations. Only scientific sources were included in this study, so the experiences of humanitarian actors using the HESPER/ HESPER Web cannot be commented on. However, even if the HESPER tool is recommended to be part of HPC and provide basis for the humanitarian response plan, it is currently not a recommended method to produce the humanitarian needs overview. Given the results of this review and the fact that HESPER can be used for both humanitarian practice and scientific use, it should be advocated also to be recommended as basis for the humanitarian needs overview. Since the psychometric evaluation of the scale is made on the whole scale, slicing the scale or using it embedded in other tools cannot be recommended. This review focused on scientific use of HESPER. To better understand the humanitarian use of the tool (interview based or web version), further studies on the practical experiences from using HESPER among humanitarians and humanitarian decision makers is needed. Also, to compare the results from different multisectoral tools used for needs assessment and their perceived feasibility is also suggested for future studies.

This review has several limitations. First, the choice of databases was limited. However, the chosen databases cover both medical and non-medical publications. Second, the review focuses only on a specific tool (HESPER/ HEPSER Web). To my knowledge, no other multisectoral tools have been evaluated and it therefore makes sense to present the results of studies that relied on this tool. According to the scoping review methodology, no structured quality assessment of the included papers was conducted. Furthermore, two of the eight papers were written by the author of this paper. Given the aim and methodology of this review, which was not to critically evaluate the findings or methodology, this circumstance is considered acceptable [30]. In addition, the limited number of included papers decreases the possibility of generalising the findings on perceived needs. However, the analysis still adds value to the methodological perspective that is the focus of this paper. One of the major limitations of this review, and an important question for future research, is how the use of the HESPER or other scientific scales to measure needs is experienced by humanitarian actors, and how information sharing between such stakeholders can be improved.

## Conclusion and recommendations

Taking an inventory of needs from the perspective of the affected population is important to tailor the response to each humanitarian emergency. The HESPER scale and HESPER Web are multisectoral tools that can be used to both determine perceived needs and indicate mental health problems in conflict and natural disaster contexts. However, the results from a scientific needs inventory should be shared with adequate humanitarian stakeholders to facilitate a proper response and foster closer collaboration between scientists, humanitarians, and the affected population. Doing so could also increase the development and use of evidence in practice when providing humanitarian aid.

## Data Availability

Data sharing is not applicable to this article as no datasets were generated or analysed during the current study.

## Abbreviations

HESPER: The Humanitarian Emergency Settings Perceived Needs Scale
HESPER Web: The Humanitarian Emergency Settings Perceived Needs Scale for Web use
